# Securing AI-Driven Systems for Environmental Sustainability in IoT Applications: A Lightweight Message-Authentication Scheme Against Telemetry Poisoning

**DOI:** 10.3390/s26196276

**Published:** 2026-10-03

**Authors:** Aala Oqab Alsalem

**Affiliations:** Department of Computer Science, College of Computer and Information Sciences, Jouf University, Sakaka 72388, Saudi Arabia; alaa.alsalem@ju.edu.sa

**Keywords:** Internet of Things, LoRa, message authentication, HMAC, replay resistance, data poisoning

## Abstract

Machine-learning models for environmental monitoring are only as trustworthy as the field measurements that train them, yet raw or custom LoRa telemetry links—unlike a correctly configured LoRaWAN network—carry no application-layer authentication, letting any in-range transmitter inject fabricated readings and poison the training pipeline. We propose a lightweight, unidirectional, encryption-free scheme in which each message carries an HMAC-SHA-256 tag over the device identifier, a timestamp, a monotonic counter, and the sensor payload; the server accepts a message only if its tag verifies and its counter exceeds a durable, transactionally updated high-water mark, giving origin authentication, tamper evidence, and duplicate or stale rejection without encryption. For an ESP32-S3/Heltec reference implementation, device–server–reference HMAC agreement was confirmed on a published test vector, with zero false accepts or rejects across a 25,000-message four-class adversarial test; both checks ran in a host-side reference implementation of the device’s algorithm, not on physical ESP32-S3 hardware. A ten-seed illustrative poisoning study on two public environmental datasets shows a value-based defence can increase PM2.5 forecast RMSE by up to about 104% by discarding genuine extremes, whereas our scheme holds the clean baseline exactly, since forged rows are rejected before training. The tag grows an example message from 34 to 99 bytes (about 2.9×) and roughly doubles airtime (about 2.2×); per-message CPU time and energy are not quantified here. Under independent per-device keys and rate limits, partial key compromise bounds admitted forged traffic to the compromised devices’ share of total traffic.

## 1. Introduction

Environmental sustainability increasingly rests on data-driven decisions—anticipating cold snaps, scheduling irrigation, forecasting air quality, balancing local energy demand—and the models behind them learn from large volumes of field measurements. A growing share of those measurements come from battery-powered wireless IoT nodes that sit untended in the field for years and therefore favour low-energy radios such as LoRa and minimal on-device computation [1,2]. The same properties that make such deployments affordable—an open shared medium and lightweight endpoints—widen their attack surface [3,4,5].

A model is no more trustworthy than its training data. On an unauthenticated raw or custom LoRa link—as distinct from a correctly configured LoRaWAN deployment, which already provides network-layer integrity and frame-counter replay protection—any transmitter in range can inject fabricated readings that the receiver processes as genuine. Once such readings reach a training set or online-learning pipeline they act as a poisoning attack, skewing the model, suppressing the events that should raise alerts, or inflating error; poisoning tailored to regression can move predictions substantially with only a small fraction of corrupted data [6,7,8].

Defending the model therefore has to begin at the sensor. Encryption alone does not settle this: confidentiality hides the payload but neither establishes who produced a message nor prevents replay, and public-key operations remain costly on the smallest microcontrollers [9,10]. What constrained telemetry needs first is cheap, verifiable authenticity and freshness from a design that is unidirectional, symmetric, and stateless on the wire apart from a small counter.

Rather than proposing a new message-authentication code (MAC), this paper asks what a correct, reproducible authentication layer for raw or custom LoRa telemetry should look like in practice: how messages are encoded, how replay is handled, what assumptions key management requires, what the implementation costs on the actual ESP32-S3 target, and how provenance enforcement changes the data entering a learning pipeline. The cryptographic ingredients are standard; the contribution is a correct, reproducible, measured reference design and audit for raw or custom LoRa telemetry, together with an illustrative poisoning study showing why provenance should be enforced before data enter the learning pipeline. Our contributions are fourfold. First, we give a precise HMAC-based authentication and freshness design for raw or custom LoRa telemetry that binds the device identifier, a timestamp, and a monotonic counter into the authenticated data, yielding origin authentication, tamper evidence, and duplicate/stale rejection under a server high-water mark. Second, we provide a corrected reference implementation on a commodity ESP32-S3 node with a DS3231 clock and a server verifier, together with an audit of implementation defects found in the original code. Third, we report the measured static code footprint compiled for the ESP32-S3 instruction-set architecture, the calculated on-air overhead of the tag, and an example transmit-energy calculation; board-level HMAC runtime and energy are not measured in this work, and no numeric CPU-cycle/energy-per-HMAC estimate is reported. Fourth, we use a motivating ten-seed poisoning illustration on two public environmental datasets, comparing no defence, value filtering, and origin authentication, to show how provenance enforcement prevents unauthenticated forged rows from entering the learning pipeline while value-only filtering can fail on heavy-tailed data. This last part is a statement about the acceptance decision, not about downstream accuracy, which still depends on the data distribution, the learner, and legitimate-but-faulty sources; forged rows are rejected before training whatever values they carry, because the acceptance decision is independent of the submitted sensor value under the uncompromised-key threat model. In short, unauthenticated data should be refused by provenance at ingestion, not cleaned by value afterwards.

## 2. Related Work

Security of constrained IoT and LoRa telemetry. Surveys of IoT security consistently find that constrained endpoints and open media leave much traffic unprotected [3,4,5,11], with man-in-the-middle techniques mapped across the stack [12]. For LoRa, several concerns should be kept apart: implementation and configuration weaknesses in LoRaWAN key management and join procedures [13]; jamming and availability attacks at the radio layer [14]; and replay under weak frame counters, which has toggled commercial Zigbee devices despite integrity codes [15]. Direct prior work on authenticating raw, non-LoRaWAN LoRa telemetry at the application layer is comparatively sparse; a recent example combines AES encryption with HMAC on a Raspberry Pi/LoRa point-to-point link for vehicular tyre-pressure telemetry [16], and lightweight hash-based watermarking has separately been proposed to give wireless sensor networks data-freshness and tamper detection without public-key cryptography [17]. Separate from these is the problem we address: application-layer authentication for raw or custom LoRa telemetry that carries no such protection at all. A correctly configured LoRaWAN network already provides network-layer integrity and frame-counter replay protection under the LoRaWAN L2 specification [18], so the gap we target is the unauthenticated raw-link case, not a deficiency of standard LoRaWAN.

Encrypting the payload is a common response, but plain (unauthenticated) symmetric encryption only provides confidentiality: it neither authenticates the sender nor detects tampering or replay by itself, and a reused deployment key weakens it further; authenticated encryption with associated data (AEAD) modes combine confidentiality with an integrity/authenticity check in one primitive, but nothing about symmetric encryption—authenticated or not—requires one shared deployment-wide key, and per-device keys are equally compatible with either approach. Asymmetric schemes and key-agreement protocols such as elliptic-curve Diffie–Hellman add bidirectional exchanges and computational load that strain the smallest nodes [9,19,20]; the NIST lightweight-cryptography effort stresses that authenticity, not only confidentiality, is first-class [10]. Recent lightweight IoT authentication and key-agreement designs continue this direction: a 2026 one-to-many authentication scheme for constrained sensors combines an ephemeral key exchange at the gateway with symmetric HMAC/AEAD operations at the sensor itself, keeping device-side cost to hashing and authenticated encryption while still supplying a game-based and symbolic security argument [21], an argument structure we adopt for our own, simpler one-way setting in Section 6.3. Keyed hashing supplies authenticity cheaply: HMAC is standardised and well-analysed [22,23], and HOTP/TOTP [24,25] derive one-time codes from a fixed shared secret via a counter or time factor. Authenticity without freshness still admits replay [15], and time-based freshness inherits the clock’s fragility [26]; a recent lightweight telemetry-integrity design for constrained IoT sensors illustrates this trade-off directly, pairing an application-layer HMAC seal with a timestamp-based freshness/replay filter and an explicit, Dolev–Yao-style symbolic analysis that distinguishes detection of non-adaptive tampering from resistance to a fully adaptive forger [27], the same category distinction (tamper detection vs. forgery resistance vs. replay) that Section 6.3 makes for the scheme in this paper. We therefore keep HMAC for authenticity but make freshness explicit through a monotonic counter checked against a server high-water mark, avoiding reliance on clock synchronisation.

Data poisoning and provenance. A small fraction of corrupted training data can materially degrade a model, from early attacks on support vector machines [6] through broad syntheses of adversarial machine learning [28] to poisoning studied specifically for regression [7,8], with provenance-based mitigations proposed for deployed pipelines [29]. A parallel line applies machine learning to IoT defence via anomaly detection at the gateway [30,31]. These act after ingestion; we take the earlier, cheaper step of refusing unauthenticated data at the boundary and use a small illustrative poisoning case study to show how provenance enforcement prevents unauthenticated forged rows from entering the learning pipeline, while value-only filtering can fail on heavy-tailed data.

## 3. Threat Model and Scope

### 3.1. Scope: Raw LoRa Versus LoRaWAN

The scheme targets raw or custom point-to-point LoRa telemetry: a transmit-only node sends application frames over a bare LoRa physical link (or a lightweight custom protocol above it) to a receiver, with no return channel and no LoRaWAN stack. This arises in minimal, energy-first deployments that use a LoRa transceiver directly rather than joining a LoRaWAN network. A correctly configured LoRaWAN deployment provides MIC-based message integrity using network-session integrity keys and replay protection through frame counters checked at the network server, with application-session keys protecting the payload primarily for confidentiality [2]. Our threat model is therefore not standard LoRaWAN; we do not claim that correctly configured LoRaWAN accepts unauthenticated fabricated frames.

Relative to LoRaWAN, the scheme has two roles. For a raw or custom LoRa link with no such protection, it supplies the missing application-layer origin authentication, integrity, and duplicate/stale-counter rejection directly, and this is the primary intended use. For a LoRaWAN deployment it is complementary rather than redundant: it provides end-to-end, application-layer authentication of the sensor payload that is independent of the network-layer MIC and session keys, so it holds if the application-layer material is provisioned separately from the LoRaWAN keys and remains meaningful across gateways or where network-layer checks are terminated before the application server. It is not intended to replace LoRaWAN’s MIC or frame-counter mechanisms where those are correctly in force, and we do not evaluate it as a fix for misconfigured LoRaWAN.

### 3.2. Monitoring Pipeline and Poisoning Vector

**Figure 1:**
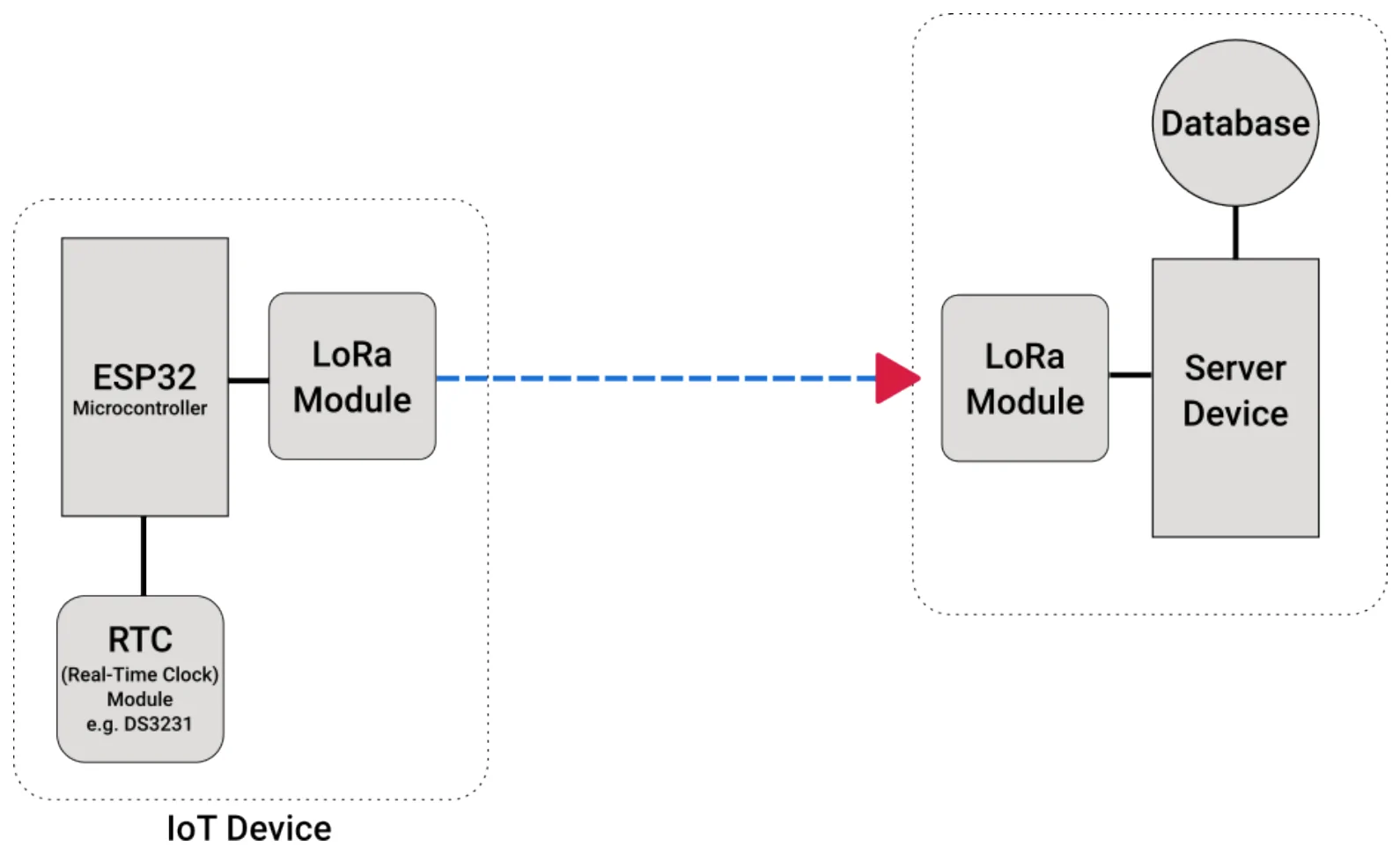
System architecture. The IoT node (ESP32-S3, LoRa module, and DS3231 real-time clock) transmits one-way to a server that verifies each message before storing it for later use by the forecasting model.

The asset we protect is the data pipeline that feeds an AI model. Each node samples an environmental variable at a fixed interval—temperature in our reference node, read from the DS3231—and transmits the reading to a gateway and server. Accepted readings are stored and later used to train or update a forecasting model, for example a one-step-ahead predictor that drives low-temperature or irrigation alerts. The pipeline is unidirectional (nodes transmit but need not receive), and its value rests on data integrity, because a poisoned training set produces a model that is confidently wrong. Poisoning here does not require breaking the model: it only requires placing plausible but false readings into the stream. An attacker able to transmit on the unauthenticated link might inject high-temperature readings to suppress low-temperature alerts, or add noise that raises forecast error. If the server rejects every message it cannot authenticate, forged readings never reach the training set, and the poison fraction seen by the model is driven to zero. Figure 1 shows the overall system.

### 3.3. Adversary Model, Goals, and Non-Goals

Choice of adversary model. The scheme is a one-way, pre-shared-key message-authentication code: there is no key exchange, no session key, and no ephemeral secret of any kind, so the eCK (extended Canetti–Krawczyk) model—designed to capture ephemeral- and static-key leakage in authenticated key-exchange protocols that negotiate a fresh session key—does not apply here; there is no session key for an eCK-style adversary to attack, and adopting eCK terminology for a stateless MAC-tag scheme would misdescribe what is actually being modelled. We instead separate three distinct layers, each with its own assumptions, and do not conflate them:

1. A Dolev–Yao (DY) symbolic communication model [32] for the wireless channel: The adversary is the network. It can observe every transmitted frame; inject arbitrary frames of its own choosing; modify, truncate, or reorder any observed frame before re-injecting it; replay a previously observed frame verbatim; and delay or drop any frame. The adversary cannot, however, construct a frame that verifies at the server without already knowing a value that hashes correctly under the per-device key *K*—within the DY abstraction, HMAC-SHA-256(K,·) is treated as an ideal, unforgeable function that the adversary cannot invert or forge without *K*.
2. A computational cryptographic assumption that justifies treating HMAC-SHA-256 this way: HMAC-SHA-256 is assumed to be a secure pseudorandom function (PRF) and hence an unforgeable MAC under standard assumptions [22,23] so that no probabilistic polynomial-time adversary without *K* can produce a fresh, valid tag except with negligible probability. Section 6.3 states this as an explicit existential-unforgeability game.
3. Implementation-level threat assumptions, which are preconditions we rely on rather than properties we prove: The per-device key *K* is generated with sufficient entropy and remains secret on both the device and the server (no physical extraction, no side-channel leakage, no server-side key-store compromise); the firmware and its persisted counter state are not tampered with; and the canonical encoding is implemented correctly on both sides. Section 6.3 states the consequences if any of these assumptions is violated (Section 4.5’s compromise model).

Concretely, under this model, the adversary within radio range of both node and gateway CAN eavesdrop on all frames; transmit arbitrary frames of its own construction; capture a legitimate frame and retransmit (replay) it, verbatim, at a later time of its choosing; reorder delivery, e.g., by delivering a captured higher-counter frame before an earlier-captured lower-counter one; and delay or drop frames outright. The adversary CANNOT, under the key-secrecy precondition above, learn *K* from observed traffic; compute a valid tag for any payload it did not observe already correctly tagged by a genuine device; or cause the server to accept a message whose counter does not exceed the stored high-water mark. This key-secrecy precondition is a precondition of every guarantee below, not a claim proven by the scheme itself; the key-management model and the consequences of its violation are treated in Section 4.5. Attacks on an IoT device can also be grouped by how close the adversary must get: physical attacks require direct access, credential attacks require discovering secrets, and messaging attacks (man-in-the-middle, spoofing, replay) can be mounted from radio range alone. This work targets the last group, where the attacker only needs to be within range. Under this model, the scheme must provide origin authentication (a message is accepted only if produced by a party holding the correct per-device secret, per the definition in Section 6.3), integrity (any modification in transit is detected and discarded), and duplicate/stale rejection (an accepted message cannot be accepted again, and any message whose counter is not strictly greater than the stored high-water mark is rejected, without clock synchronisation), at low added flash, memory, computation, and energy.

Confidentiality and availability are explicit non-goals. The scheme does not prevent jamming, dropping, delaying, or selectively reordering traffic. An attacker who controls delivery timing can cause data loss—for example, by delivering a captured higher-counter message first, making intervening legitimate messages stale—but cannot thereby inject any message the device did not produce and authenticate/tag. The counter is an integrity and freshness control, not an availability guarantee. This also means the scheme does not prevent availability-based alert suppression: an attacker who jams, drops, delays, or selectively reorders valid higher-counter messages can cause missing readings and thereby suppress or degrade alerts without forging data. The poisoning experiment studies forged-data admission, not missing-data attacks caused by radio-layer denial of service or counter-state advancement. The scheme likewise does not claim resistance to physical key extraction, side-channel analysis, or jamming; Section 6.3 and Section 7 restate these boundaries explicitly alongside the formal argument.

## 4. Proposed Authentication Scheme

### 4.1. Message Format and Verification

Before deployment, each node is provisioned with a unique identifier (DEVEUI), a per-device secret key *K* that is independent across devices, and the hash and time parameters. It also keeps a monotonically increasing counter *N* in non-volatile storage. For every reading the node builds a canonical payload and its authentication tag,

(1) P=DEVEUI‖“|”‖TIMESTAMP‖“|”‖N‖“|”‖SENSOR,

(2) TAG=HMAC-SHA-256(K,P),

where *P* is a canonical UTF-8 text string whose field encodings and delimiter are fixed in Section 4.3, and the HMAC is computed over the raw bytes of that string. The node transmits a five-section delimiter-separated line.

(3) MESSAGE=TAG‖“|”‖P,

Upon receipt, the server splits the line on the delimiter, parses DEVEUI, looks up *K* and the stored high-water mark, reassembles *P* from the four payload sections exactly as the device built it, recomputes the tag over the same bytes, and accepts the message only if the tag matches under a constant-time comparison and *N* exceeds the stored high-water mark; it then advances that mark. Binding DEVEUI stops an attacker from relabelling a captured message as another device. Because the tag is keyed by *K*, which never leaves the device or the server, an attacker without *K* cannot forge a valid tag for any chosen payload.

**Figure 2:**
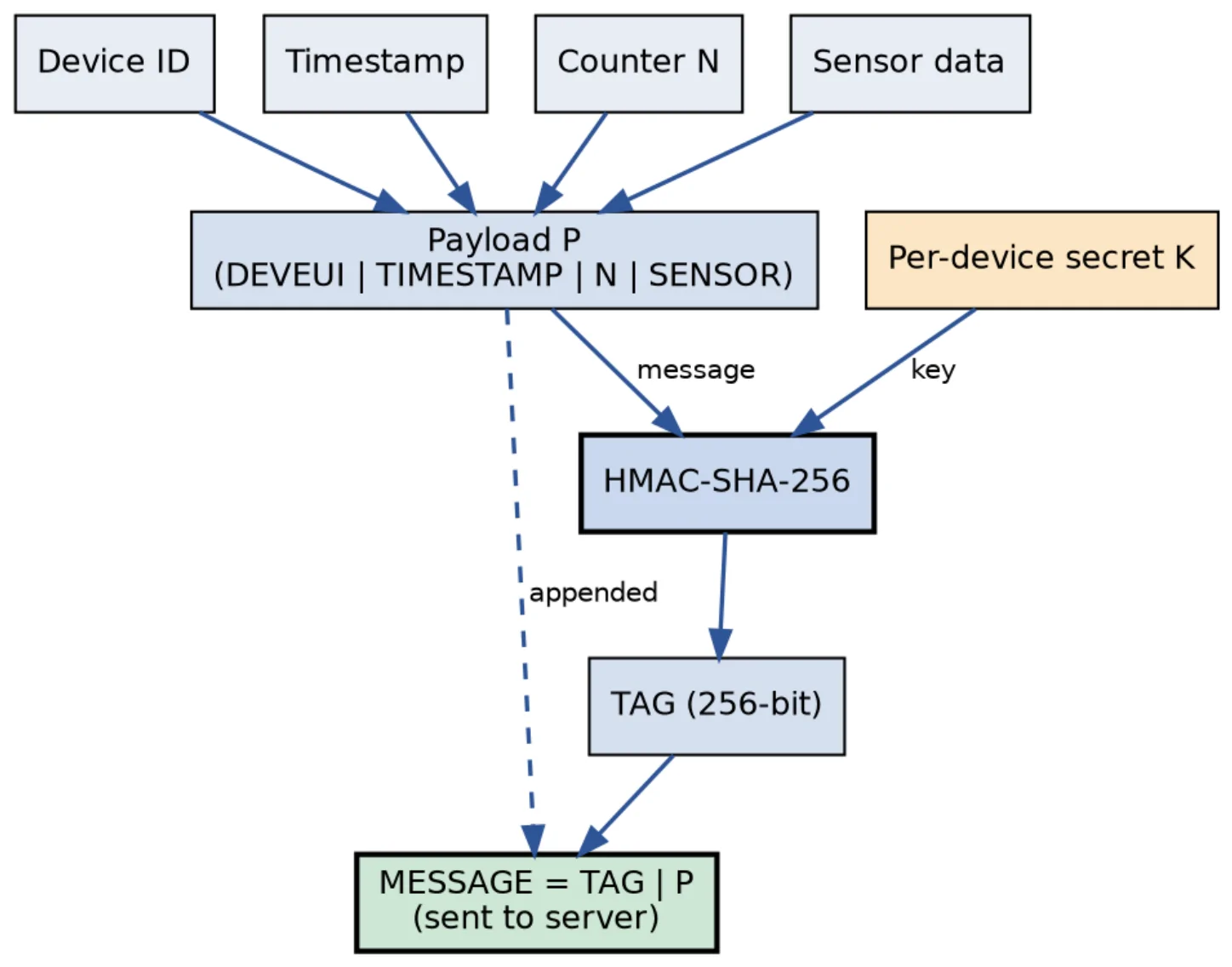
Construction of an authenticated message. The device identifier, timestamp, counter, and sensor data form the payload *P*; the tag is the HMAC-SHA-256 of *P* keyed by the per-device secret *K*; the transmitted message is the tag followed by *P*.

Freshness depends solely on the monotonic counter and its high-water-mark check. The timestamp is bound into the authenticated payload so that any tampering with it is detected, and it is available for logging and coarse sanity checks, but the server does not use it in the acceptance decision and therefore does not rely on clock synchronisation. Figure 2 shows how one authenticated message is assembled.

### 4.2. Protocol Flow and Reordering Behaviour

**Figure 3:**
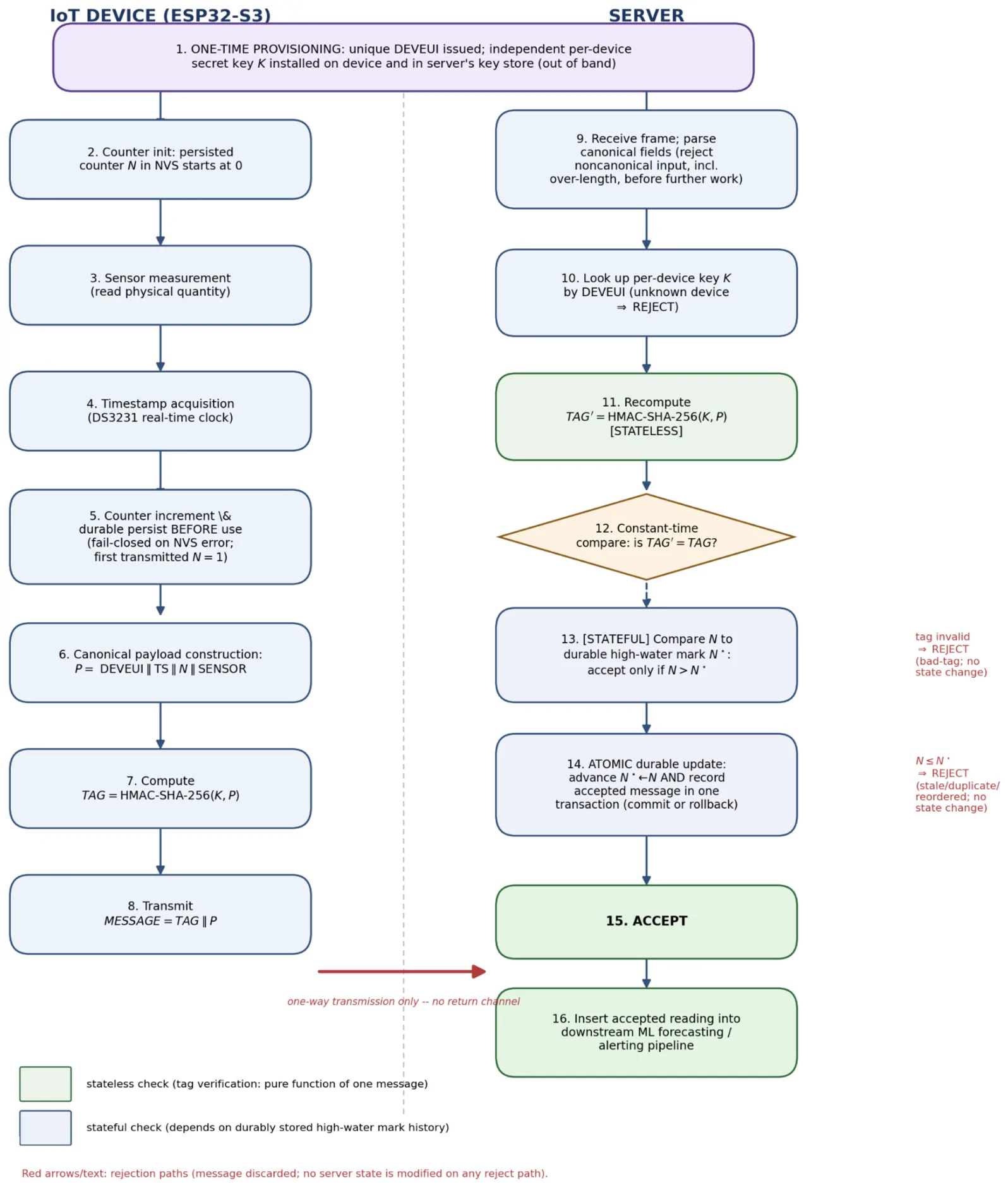
Complete one-way protocol flow. **Left**: device-side steps (one-time provisioning; counter initialisation; per-reading sensor/timestamp acquisition; counter increment and durable persistence; canonical payload construction; HMAC-SHA-256 tagging; transmission). **Right**: server-side steps (parsing; key lookup; stateless tag recomputation and constant-time comparison; stateful high-water-mark comparison; atomic durable state update; accept/reject; and, on acceptance, insertion into the downstream ML pipeline). Red text marks the two independent rejection paths, neither of which changes server state.

Figure 3 gives the complete end-to-end protocol flow, from one-time provisioning through to insertion of an accepted reading into the downstream machine-learning pipeline. The scheme is explicitly unidirectional: the node transmits and never receives, so the diagram deliberately contains no return channel, no challenge–response exchange, and no random-state dispatch from the server back to the device—any such step would require a receiver on the node and a second radio link, neither of which this design uses. The diagram separates the two acceptance checks by statefulness, matching the distinction introduced in Section 3: tag verification (steps 9–12) is a stateless function of the single received message, whereas the counter check (steps 13–14) is stateful, depending on every message the server has previously accepted from that device, and is applied only after the stateless check has already passed.

Reordering, high counters, and why this is not a forgery. A reviewer raised a specific and important scenario: an attacker who intercepts a legitimate message carrying a high counter value and forwards it to the server before an earlier, lower-counter message is delivered. Under the accept rule of Section 4.5 ($N>N^{★}$), the high-counter message is indeed accepted, and the server’s high-water mark advances to that value; the lower-counter message, when and if it later arrives, is then rejected as stale, even though it too was genuinely produced by the device. This is expected, intended behaviour, not a gap in the scheme, and it follows directly from keeping authentication and freshness as two separate properties: authentication asks “was this payload produced by a party holding *K*?” and is answered by the tag check alone; freshness asks “has a message with this counter value already been accepted?” and is answered by the high-water-mark check alone. A message can be authentic without being fresh (a valid replay) and the scheme’s high-water mark exists precisely to catch that case; what it does not do, and does not claim to do, is guarantee in-order delivery of authentic messages. Table 1 works through the reviewer’s scenario explicitly.

Two points follow directly from Table 1. First, the attacker never causes the server to accept a value the device did not produce: step 4 shows that attempting to fabricate or alter a sensor reading, at any counter value, fails the tag check regardless of the counter’s freshness, because the tag is keyed by *K* and the attacker does not have it. Second, the cost of the reordering attack falls entirely on availability, not on integrity: readings N=10 and N=11 are lost to the training pipeline (a missing-data event, addressed by monitoring or retransmission at the application layer, both out of scope here), but no fabricated or modified value is ever accepted. The scheme therefore does not provide message ordering, delivery guarantees, or recovery of skipped legitimate messages; Section 7 restates this as an explicit limitation, and Section 6.3 restates it within the formal argument.

### 4.3. Canonical Encoding

Simple concatenation is well defined only once the byte representation of every field is fixed, so both the firmware and the server implement the canonical encoding of Table 2. The payload *P* is a UTF-8 text string whose four fields are joined by the single-byte ASCII delimiter “|” (0x7C) in a fixed order; because every field is hexadecimal or a decimal number, the delimiter cannot occur inside a field and no escaping rule is required. The HMAC is computed over the raw UTF-8 bytes of *P*. The cryptographic tag is 32 bytes (256 bits), transmitted as 64 lowercase hexadecimal characters (64 ASCII bytes on the wire). Before HMAC verification, the server rejects noncanonical encodings (wrong field count, non-lowercase-hex tag or identifier, zero-padded or out-of-range integers, whitespace, or non-ASCII bytes) and reconstructs the payload only from validated fields, so a representation that is not byte-identical to the canonical form can never be accepted even if it would hash to a valid tag under a looser parse. The full parser rules are provided in the Supplementary Materials.

The canonical wire FORMAT places no bound on *TIMESTAMP* beyond the unsigned-64-bit range, so TIMESTAMP = 0 is syntactically well-formed and is accepted by the parser; whether a deployed node would ever legitimately transmit it is a separate, deployment-level question (for example, an RTC that has lost backup power might read back epoch 0 on next boot). Because the server’s acceptance decision depends only on the counter and never on the timestamp value (Section 4.5), accepting *TIMESTAMP* = 0 at the format layer has no bearing on freshness or replay rejection. The *SENSOR* range in Table 2, by contrast, is enforced by the reference server’s parser as a deployment policy choice reflecting a plausible physical range for the reference use case; it is not dictated by the canonical encoding itself, and a different deployment could set a different range or none at all.

The scheme was validated against a fixed test vector: for the canonical payload 70b3d57ed0000001|1721390400|42|235 under a 32-byte key, the device HMAC library, the server’s PHP hash_hmac, and an independent reference all reproduce the tag d5fe9b5e…a7ffa56b, confirming that all three hash byte-identical input. The device library additionally reproduces the published RFC 4231 HMAC-SHA-256 test vectors [33], though passing published vectors does not by itself rule out edge-case defects. The complete byte-level test vector and the test-vector scripts are in the Supplementary Materials.

### 4.4. Why Not a TOTP-Derived Key

The original design used a TOTP-style value as the key for the payload tag, changing it each time window. This conflates the long-term per-device secret *K* with a one-time code derived from it: a TOTP value is an authentication code, not a high-entropy replacement for the secret. A short decimal TOTP value must therefore not be used directly as the HMAC key—six digits is roughly twenty bits, so an attacker who captures a single message can search the code offline within the window and forge messages. If per-window key separation is desired as defence-in-depth, derive a full-entropy window key from *K* with HKDF [34], placing the window index in the HKDF-Expand info context rather than substituting it for the salt; the derived window key, not a TOTP code, is then used as the HMAC key. Our design keeps the long-term secret *K* as the HMAC key directly and makes freshness explicit through the monotonic counter, so no derived window key is required. The HKDF construction is given in full in the Supplementary Materials.

### 4.5. Counter Rule and Key Management

Counter and acceptance rule. The counter *N* is a 32-bit unsigned integer, initialised to 0 and stored in the device’s non-volatile storage (ESP32 NVS). For each reading, the firmware reads the stored value, increments it, and writes it back before transmitting, so a transmitted counter is never smaller than one already persisted and the very first transmitted message from a fresh device carries N=1; a power loss after persisting but before transmission merely skips a value. The server stores a per-device high-water mark $N^{★}$ (initialised to 0 for every device, not left unset) and accepts a message iff its tag verifies and $N>N^{★}$, and it then sets $N^{★}\leftarrow N$. Because $N^{★}$ starts at 0, a first message carrying N=0 is rejected exactly like any other stale counter, from the very first message a device ever sends—there is no special-cased “first message from an unseen device” exception. It accepts any $N>N^{★}$, not only $N=N^{★}+1$, so the channel does not assume in-order delivery and skipped counters are tolerated; duplicates and stale counters are rejected, while reordering (including an attacker who delivers a captured higher-counter message first) can still cause data loss but never forgery. On rollover after $2^{32}-1$ messages, or after a lost counter or cloned device, the device would be re-provisioned with a fresh key and both counters reset to 0 at the same time (the key-management paragraph below states precisely which part of this the prototype actually implements); because the key changes, previously captured messages cannot be replayed, and because the counter reset and the key installation are applied together as a single re-provisioning step at both the device and the server, there is no window in which the old key is retired but the old high-water mark is still live (or vice versa). NVS write endurance, rather than the counter space, is the binding lifetime constraint for high-rate nodes; an order-of-magnitude endurance analysis and mitigations are in the Supplementary Material.

Message acceptance is a two-part decision with different statefulness: the HMAC tag/format check is stateless (it depends only on the message just received and the device’s stored key), while the counter/high-water-mark check is stateful (it depends on every message previously accepted from that device). The reference server implementation reflects this split directly: a stateless canonical-parser-and-tag verifier is a pure function of one message, and a separate stateful store applies the high-water-mark rule on top of it. Because acceptance is stateful, the high-water-mark update and the durable record of the accepted message must be committed together: our reference stateful store performs both inside a single database transaction, so a crash between “the tag verified and the counter is fresh” and “the mark was advanced” cannot leave the two out of step— either both effects are committed or neither is, and a message is never treated as accepted without a corresponding durable state update.

Key management. Each device is provisioned with an independent 256-bit secret key *K*; no deployment-wide shared key is used, so compromise of one device key does not reveal others. In a deployment, keys are generated by a CSPRNG during provisioning and written, unique per device, into protected device storage and into the server’s per-device key store indexed by DEVEUI. The reference implementation instead uses placeholder keys for reproducibility—a fixed device constant in firmware flash and a matching plaintext server entry—and does not use secure boot, flash encryption, encrypted or eFuse-protected NVS, or a secure element. It therefore offers no resistance to physical key extraction; deployed systems must provision CSPRNG-generated secrets into protected storage (eFuse or a secure element on the device; an HSM, KMS, or at least an encrypted store on the server) and enable the platform’s secure-boot and flash-encryption features. Cryptographic key rotation is an operational requirement that is NOT implemented in this prototype. In a deployment, revoking a device would work by having the operator add its DEVEUI to a denylist at the server, after which no message bearing that identifier is accepted; rotating a device’s key would work by installing a new key and resetting both the device’s and the server’s counter state atomically, as one re-provisioning step, so no window exists in which the old key is retired but the old high-water mark is still live, or vice versa. Neither the denylist nor cryptographic key rotation is implemented in the reproducibility archive: the prototype implements and tests only the counter-STATE side of re-provisioning (the high-water mark resetting to 0 so the next message must again carry N≥1), against a server whose key lookup is a single static key per DEVEUI with no version parameter and no revocation list. A deployment implementing either mechanism would benefit from the stateless tag/format check requiring no round-trip to the device, so revocation or rotation could take effect immediately from the server’s next received message; this is a property of the design that a deployment could exploit, not a capability demonstrated by this prototype.

Compromise model. The origin-authentication guarantee holds only while the per-device key remains secret and the firmware and counter state are intact. If an attacker extracts *K* from a device or the server store, that device’s identity is fully compromised: the attacker can produce messages indistinguishable from the genuine device, because cryptographically they are that device. Nothing in the scheme recovers from this beyond revocation; the residual protection is containment of scope to the affected device, quantified in Section 6.3.

## 5. Implementation and Cost

The reference node is a Heltec WiFi LoRa 32 (V4) [35]: an Espressif ESP32-S3R2 [1] (dual-core Xtensa LX7 up to 240 MHz, 512 KB SRAM, 2 MB PSRAM, 16 MB flash) with a Semtech SX1262 transceiver and an Adafruit DS3231 real-time clock [36]; the server is a PHP verifier. All static-cost measurements target this instruction-set architecture (ISA); no board-level measurement was taken (the compiled object was never flashed to or timed on physical hardware for this revision). The ESP32-S3 has on-chip SHA/HMAC accelerators, but we compute HMAC-SHA-256 in software; the reported path is software-only and the hardware-accelerated path is not evaluated. Auditing the original firmware and server corrected four implementation defects that had to be fixed before any reported cost was meaningful: the C bitwise-XOR operator (⌃) used where exponentiation was intended (collapsing a six-digit value to about 3.6 bits), inconsistent tag truncation between device and server (a genuine message failing verification), noncanonical width-padded field formatting (changing the hashed bytes), and instruction counting via AVR disassembly on an Xtensa target. The corrected implementation adopts the canonical encoding of Section 4.3, compares tags in constant time (hash_equals), and keeps deployment secrets out of source; the reproducibility archive uses explicitly marked non-deployment test keys (a fixed, publicly documented value used only to reproduce the test vector below), which necessarily do appear directly in the archive’s source files precisely because they are not deployment secrets. Details of each defect are documented in the Supplementary Materials.

### 5.1. Static Footprint and On-Air Cost

We separate two quantities that are on a firm empirical footing: the static size of the compiled authentication code, which we measure; and the on-air cost of transmitting the tag, which follows exactly from the published LoRa airtime formula and the fixed message lengths. A third quantity—per-message CPU runtime and energy—would require either a board measurement or a cycles-per-byte figure benchmarked specifically for the ESP32-S3’s Xtensa LX7 core running this exact software SHA-256 implementation; we were unable to locate a citation meeting that bar (see the note at the end of this subsection), so runtime cycles and energy are not reported as numeric estimates in this revision and are left to future board-level measurement.

Static disassembly counts describe compiled-code size, not instructions executed per message. We compiled the SHA-256/HMAC translation unit with the ESP32-S3 Xtensa toolchain (target elf32-xtensa-be, i.e., 32-bit Xtensa ELF objects, big-endian) and read section sizes with size and the instruction count from objdump; the disassembled count is 540 Xtensa instructions over the SHA-256/HMAC symbols (of which hashBlock is 145) in a 1572-byte .text. These are properties of the binary; the exact commands and saved outputs are in the Supplementary Materials. One HMAC over the canonical payload performs exactly four SHA-256 compression-function invocations—the inner hash over the (K⊕ipad) block plus the padded payload (two blocks) and the outer hash over the (K⊕opad) block plus the 32-byte inner digest (two blocks)—so 4×64=256 bytes pass through compression, independent of small payload-length variation in this range; this count is derived directly from the HMAC/SHA-256 constructions and is not an empirical estimate.

On the wire, the unauthenticated payload is 34 bytes and the authenticated message 99 bytes. The added 65 bytes break down as: the 32-byte raw HMAC-SHA-256 tag, hex-encoded to 64 ASCII bytes on the wire (32 bytes of hex-encoding overhead beyond the raw tag), plus 1 delimiter byte joining the tag to the payload. The authenticated message is therefore 99/34≈2.91× the unauthenticated length, a 191% increase—not a doubling; transmitting the tag as 32 raw binary bytes rather than 64 hex characters would remove the 32-byte hex-encoding overhead and bring the total to 67 bytes (≈1.97×, a 97% increase), without changing the tag length or security margin; truncating the tag would reduce the on-air overhead further but weakens the online guessing bound to $2^{-t}$ for a *t*-bit tag and is a separate design choice governed by risk tolerance. At SF7, 125 kHz bandwidth, coding rate 4/5, 8-symbol preamble, explicit header, and CRC on, the LoRa airtime formula gives 77.1 ms for the payload and 169.2 ms for the authenticated message, an incremental 92.2 ms; the authenticated message’s airtime is therefore 169.2/77.1≈2.20× the unauthenticated airtime, a 120% increase—close to, but not exactly, a doubling, and numerically distinct from the 191% byte-length increase because airtime includes a fixed preamble/header cost that does not scale with payload length. Transmit energy follows $E_{\mathrm{TX}}=V\cdot I_{\mathrm{TX}}\cdot t_{\mathrm{air}}$; the SX1262’s manufacturer-specified transmit current spans 32–118 mA across its output-power range [37], with the lower end of that range obtained under DC-DC supply regulation and the upper end (118 mA) specified at +22 dBm under battery supply—DC-DC regulation draws less current than LDO regulation at a given output power per the datasheet’s power-distribution guidance. At the example $I_{\mathrm{TX}}=32$ mA, V=3.3 V point, the tag adds about 9.7 mJ. Table 3 summarises these costs.

For context, the working-memory footprint of this keyed-hash path is small in absolute terms: the measured/estimated figures above (.text, .rodata, and stack) sum to under 2.5 KiB, well within the ESP32-S3’s 512 KB SRAM. We do not offer a quantitative comparison against public-key alternatives such as elliptic-curve Diffie–Hellman in this revision: a prior reported ECDH working-memory figure for constrained devices [20] was measured on a different software stack, toolchain, and (in part emulated) hardware platform than the one used here, so a numerical ratio between the two would not be like-for-like. Qualitatively, symmetric HMAC avoids the elliptic-curve group operations and larger key/point representations that key-agreement protocols require, and the NIST lightweight-cryptography effort motivates exactly this kind of preference for symmetric authenticity primitives on the smallest devices [10]; a fair quantitative comparison would require benchmarking both constructions on the same board, toolchain, and methodology, which we leave for future work. Board-level HMAC timing and energy also remain future work; the static measurement is preserved as a saved build artifact, with the measurement commands and a disassembly excerpt provided in the Supplementary Materials, but the exact Arduino-linked build inputs needed to reproduce the 1572-byte figure byte-for-byte are not included, so a user recompiling only the archive’s portable source should not expect to regenerate that exact value (the archive documents this explicitly and reports the different, smaller size obtained from the portable build for comparison).

Note on unsupported numeric estimates. An earlier version of this manuscript reported a CPU runtime/energy estimate for the HMAC computation derived from a “published software SHA-256 rate of 25–40 cycles/byte” and an assumed “30–50 mW active core power,” without a specific, architecture-matched citation for either figure. We were unable to identify a primary source establishing those specific numbers for software SHA-256 on the ESP32-S3’s Xtensa LX7 core, and generic cycles-per-byte figures for SHA-256 vary substantially across architectures, compilers, and implementations in the wider literature we surveyed. Rather than retain an uncited numeric assumption, we have removed the CPU-cycle and CPU-energy-per-HMAC estimates from this revision; the SHA-256 compression-block count (a derived, non-empirical fact) is retained, and a board measurement is identified as the appropriate way to establish these figures in future work.

### 5.2. Comparison with Existing Authentication Approaches

Table 4 places the proposed scheme’s overhead alongside three points of reference: LoRaWAN’s built-in network-layer integrity mechanism, a representative public-key (ECDH) key-exchange scheme, and generic authenticated encryption with associated data (AEAD). We report only values that are measured or calculated in this work or that are stated in an authoritative specification or a cited source; where no comparable figure is available, the cell says so explicitly rather than presenting an estimate as if it were measured. Consistent with Reviewer 2’s correction (Section 5.1), no CPU-cycle or power figure is reintroduced without an architecture-matched source, and the previously withdrawn two-orders-of-magnitude ECDH memory comparison is not restated.

Two qualitative observations follow without requiring a disputed quantitative claim. First, LoRaWAN’s MIC and frame counter solve a related but distinct problem: they protect the integrity and freshness of a frame between a device and a network server that is part of a correctly configured LoRaWAN deployment (Section 2), whereas this scheme targets raw or custom point-to-point LoRa links that have no such network-layer protection at all; the two are complementary rather than substitutable, as already stated in Section 2. Second, generic AEAD and our HMAC-only design share the same underlying cost driver—a single symmetric-key pass over the payload—so we would not expect a qualitatively different transmission or CPU cost from switching primitives; AEAD’s additional confidentiality guarantee is a capability this scheme deliberately does not provide (Section 3), not a cost this scheme avoids by omission. A fair quantitative benchmark of HMAC-SHA-256 against AES-GCM or a specific ECDH curve on the same ESP32-S3 board, toolchain, and methodology used here is identified as future work (Section 7).

## 6. Experimental Evaluation

We report two experiments: an implementation sanity test of the accept/reject logic and a motivating ten-seed poisoning illustration comparing no defence, value filtering, and origin authentication. Both use public datasets and deterministic seeded procedures; the clean series are real environmental datasets and only the adversarial poisoned rows are synthetic. Scripts and raw outputs are provided in the Supplementary Materials submitted with this manuscript.

### 6.1. Attacker Experiment

This experiment checks that the corrected verifier applies its accept/reject logic correctly on representative inputs; it is not an empirical proof of cryptographic security, which follows from the HMAC assumptions in Section 6.3. Using the corrected scheme, we generated 5000 genuine messages from a registered device and 5000 messages in each of four adversarial classes produced without the secret *K*: a random 256-bit tag; a valid payload signed with a wrong key; a captured message whose sensor value is altered but whose tag is kept; and an exact retransmission of a delivered message. The server accepted all genuine messages and rejected all adversarial messages (Table 5): 0 false accepts in 20,000 adversarial messages and 0 false rejects in 5000 genuine ones. These are outcomes on a finite test set, not an estimate of the cryptographic forgery probability, which follows from the HMAC assumptions of Section 6.3.

### 6.2. Data-Poisoning Experiment

If the server discards every unauthenticated message, no poison reaches the model, and accuracy is trivially preserved. To make the comparison substantive, we test authentication against the natural alternative—a defence that inspects the values rather than the origin of incoming data—and let the attacker adapt to it. The question is not whether authentication works but whether it offers anything a good data-cleaning defence does not.

#### 6.2.1. Setup

**Figure 4:**
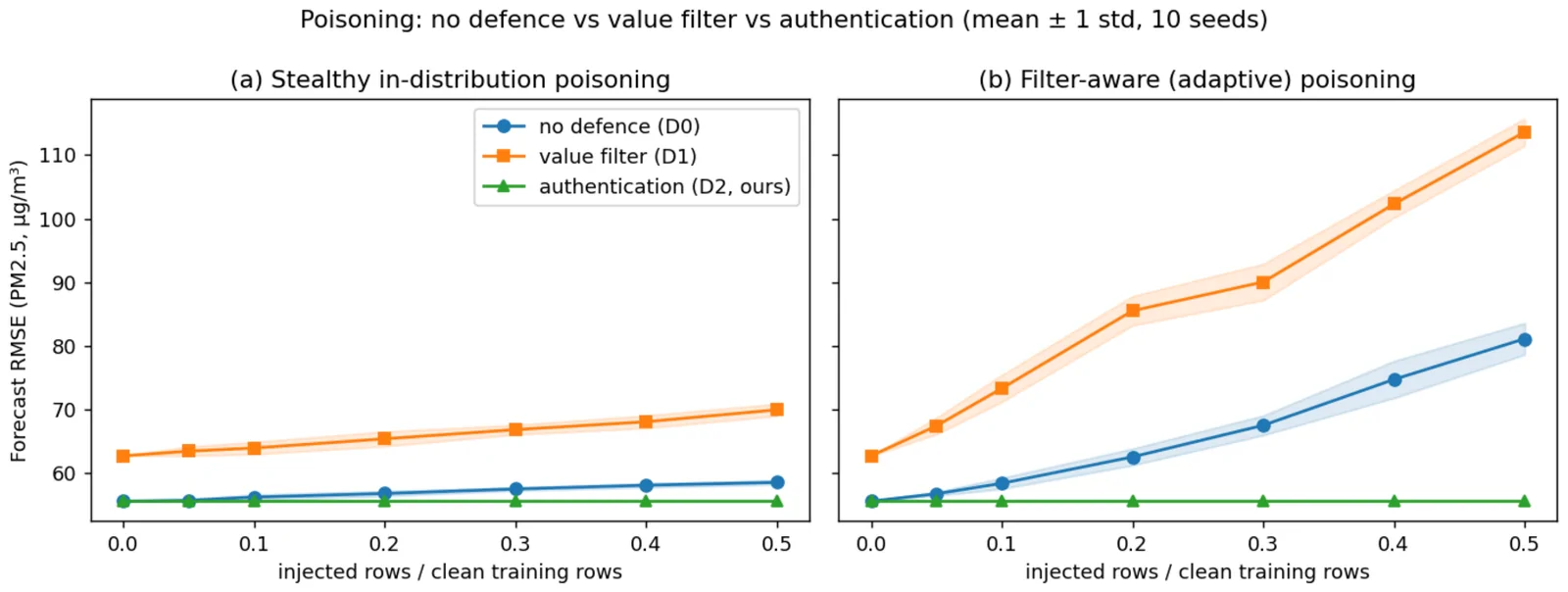
Forecast RMSE on the real PM2.5 series under (**a**) stealthy in-distribution poisoning and (**b**) filter-aware poisoning for three server postures; mean ± 1 standard deviation over ten seeds (shaded band). The no-defence model is now measurably (not negligibly) affected under the corrected, window-propagating poisoning pipeline, the value filter worsens accuracy further by discarding genuine pollution spikes, and authentication holds the clean baseline exactly throughout.

**Figure 5:**
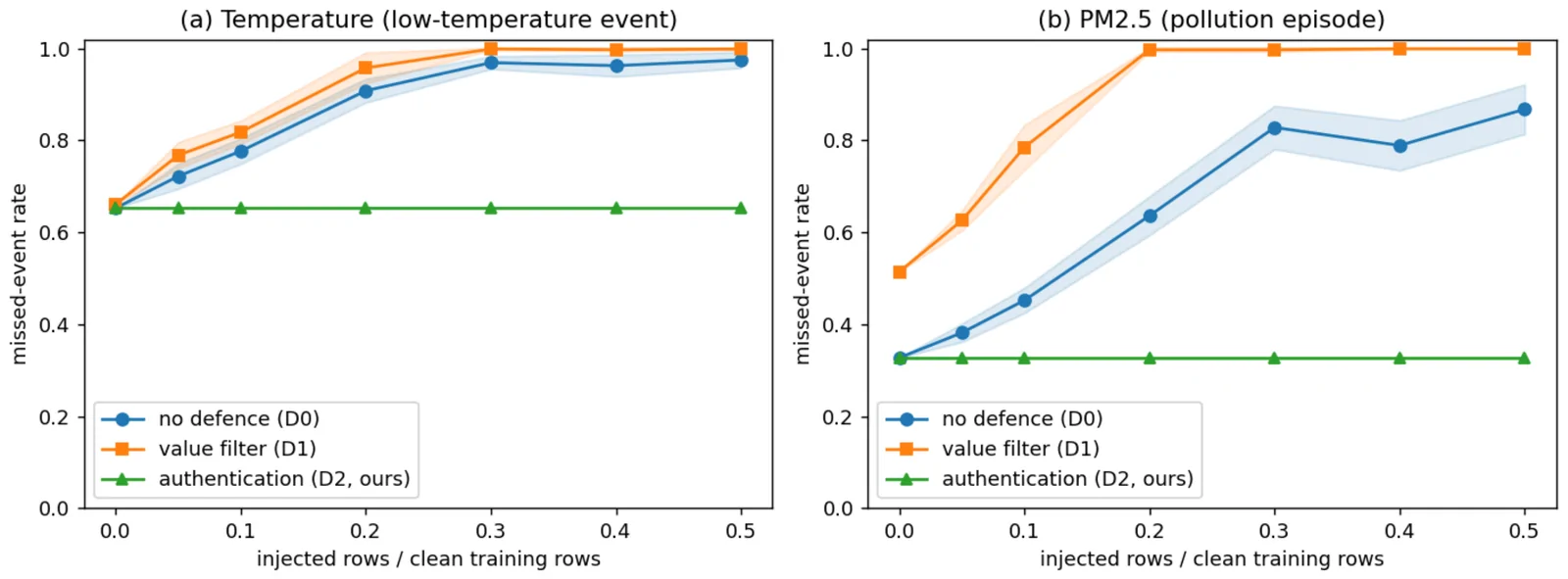
Missed-event rate under targeted alert-suppression attacks on (**a**) temperature (low-temperature event, below the 15th percentile of the training data) and (**b**) PM2.5 (pollution episode, above the 85th percentile of the training data); mean ± 1 standard deviation over ten seeds (shaded band). The robust value filter rises above the no-defence case at every matching poison fraction because trimming removes genuine extreme events; authentication holds the miss rate at its clean value throughout.

We use two public environmental series of different character: daily minimum temperatures in Melbourne, 1981–1990 (3650 observations) [38,39], and hourly Beijing PM2.5 readings, 2010–2014, subsampled to six-hourly [40,41] (downloaded as pollution.csv, SHA-256 892e9559205d16d32623135c127a3951c12e46ea1e0b3093e107ce89a9fd60e2; the temperature series is downloaded as daily-min-temperatures.csv, SHA-256 8b9de63ed6789492bf497625e7f9beb96a63d367b4b0a21754006f749fa5e5da). Each task forecasts the next value one step ahead from the seven preceding observations (a seven-lag window; seven days for temperature, 42 h for six-hourly PM2.5), augmented with a day-of-year (sin,cos) seasonal encoding computed from each observation’s real calendar timestamp (parsed from the dataset’s own date/time columns), not from its array index; an index-based encoding is only correct when one array step equals one day; for the six-hourly PM2.5 series it previously gave a 91.25-day period instead of one year, which we correct in this revision. The learner is scikit-learn’s HistGradientBoostingRegressor [42] (scikit-learn 1.8.0, Python 3.12.3, NumPy 2.4.4, pandas 3.0.2), with max_depth = 4, max_iter = 200, learning_rate = 0.06, and random_state set to the run seed; no input normalisation is applied (the model is scale-invariant). The chronological split (first 70% train, last 30% test, no shuffling, no separate validation split) is applied to the raw series before any statistic is computed, giving 2550 training/1093 test windows for temperature and 5107 training/2190 test windows for PM2.5; its clean RMSE is 2.25 °C (temperature) and $55.6\;\mu g/m^{3}$ (PM2.5), both means over ten seeds with negligible (<0.01) seed-to-seed spread. Every statistic used to craft an attack or set the alert threshold—percentiles, standard deviation, target median/MAD, and the threshold itself—is computed from the training portion of the raw series only, never from the held-out test data. We compare three server postures: no defence (D0); a robust value filter that drops targets with median-absolute-deviation *z*-score above 3.0 and refits after one residual-trimming pass (D1); and authentication (D2), under which forged rows never enter training. The attacker injects forged readings using an in-distribution stealthy profile and a filter-aware profile placed inside D1’s acceptance region; a targeted alert-suppression profile places poison just on the non-event side of the alert threshold. Each forged reading is inserted at an explicit position in the chronological training stream, with an interpolated timestamp so its seasonal feature is genuine rather than zero-padded, and every lagged forecasting window is rebuilt from the resulting mutated stream, so a single forged reading contaminates every window whose seven-lag history includes it. The injection ratio is ϕ=k/n with k=⌊ϕn⌋ forged readings inserted into *n* clean training rows over ϕ∈{0,0.05,0.1,0.2,0.3,0.4,0.5}. The full value-filter and attack definitions, hyperparameters, seeds, and raw result tables are in the Supplementary Materials. The results are the mean over ten independent seeds (20260423–20260432, varying both the attack-construction RNG and the model’s random_state), with shaded bands in Figure 4 and Figure 5 showing ±1 standard deviation across seeds; the percentages below are relative to the clean no-poison baseline on the same test split.

Defense Can Backfire—And the Corrected Pipeline Shows Poisoning Itself Is More Damaging than Previously Reported.

Rebuilding lagged windows from the mutated chronological stream (rather than appending poisoned rows in isolation with zero-padded seasonal features) lets a forged reading contaminate every window whose seven-lag history includes it, so poisoning is measurably more damaging under this corrected pipeline than under the previous isolated-row version, for every defense, including no defense at all. On the heavy-tailed PM2.5 series, the value filter remains counterproductive: genuine pollution spikes look like outliers, so trimming discards them, and the mean RMSE at ϕ=0.5 rises relative to the clean baseline by about 26% under the stealthy profile and about 104% (roughly a doubling) under the filter-aware profile (Figure 4). The no-defence model is no longer negligibly affected under the corrected pipeline—it also rises, by about 5% (stealthy) and 46% (filter-aware) at ϕ=0.5—but the value filter is worse than no defence at every matching poison fraction on PM2.5 and worse on temperature as well (about 4–5% stealthy, 5–47% filter-aware for no-defence, vs. comparable-to-larger increases under the value filter; full per-fraction means and standard deviations over ten seeds are in the Supplementary Materials). Authentication holds the clean baseline exactly, for every seed, because it never inspects values and never trains on a forged reading. This illustrates a general risk of value-based defences: they can be steered by an attacker who understands the filter, or can harm performance on heavy-tailed data by removing genuine extremes, and now that poison propagates realistically through overlapping lag windows, the same forged readings measurably degrade even an undefended model. In contrast, under the uncompromised-key threat model, the authentication decision does not depend on the submitted sensor value.

#### 6.2.2. Targeted Alert Suppression

The gap is sharpest when the attacker pursues an operational goal. Suppose the system raises an alert for an extreme event—a low-temperature event (temperature below the 15th percentile of the training data, a distributional rather than meteorological threshold) or a pollution episode (PM2.5 above the 85th percentile of the training data)—and the attacker wishes to suppress it. On both series, the value filter is not merely ineffective but counterproductive: its trimming discards genuine extreme observations while retaining the threshold-aware poison, so the missed-event rate rises above the no-defence case at every matching poison fraction, whereas authentication pins the miss rate to its clean value (Figure 5). Both alert tasks are intrinsically hard for a one-step forecaster predicting near the conditional mean—the clean model misses about 65% of low-temperature events (121 events in the 1093-row test split) and 33% of high-PM2.5 episodes (278 events in the 2190-row test split)—so the quantity of interest is the relative effect of the defense; for PM2.5, the filter raises the mean miss rate from about 0.33 (clean) to a range of about 0.51–1.00 across poison fractions, while no defense at all raises it to about 0.38–0.87 over the same range—both rise well beyond the clean rate, and the filter is consistently the worse of the two. Per-fraction rates, standard deviations, and confusion counts are in the Supplementary Materials. The attack studied here is forged-value admission. A separate availability attacker could also suppress alerts by preventing genuine readings from arriving; that case is outside the authentication guarantee and requires redundancy, monitoring, retransmission, or operational fail-safe logic.

#### 6.2.3. Containment Under Key Compromise

The claim we make under partial compromise is cryptographic containment: with independent per-device keys, an attacker holding the keys of *c* of *N* devices can authenticate only as those *c* devices, so under equal-rate devices and per-device rate limits, the admitted forged traffic is bounded by c/N; without those controls a faster compromised node could enlarge its share. We do not claim graceful accuracy degradation: the mean RMSE rises mildly and roughly monotonically with the compromised share under the corrected pipeline (temperature 2.25→2.32, PM2.5 55.6→58.6, means over ten seeds, from 0 to 10 of 20 compromised devices)—a directional change from the previously reported flat-to-slightly-decreasing pattern, though the magnitude remains modest: at most a 3% (temperature) to 5% (PM2.5) increase over the clean baseline, a small fraction of the 46–104% increases seen under a full, undefended poisoning campaign at a comparable overall injection ratio (Figure 4). This shows no accuracy collapse over the tested range; the protection that holds is the traffic-share bound, not graceful degradation of accuracy. Across the two dissimilar series the pattern was consistent, addressing single-dataset external-validity concerns, though not across model families since only the gradient-boosted learner was evaluated.

### 6.3. Security Analysis

We state the guarantees against the adversary of Section 3. Here, origin authentication means authentication of a message as originating from a holder of the registered per-device secret under the stated key-secrecy assumption; it is not a claim about the physical device’s identity independent of that secret; a party who has extracted or been issued the secret is indistinguishable from the registered device under this definition. Let M=t‖“|”‖P with t=HMAC-SHA-256(K,P) and *P* the canonical payload of Section 4.3; the server accepts *M* iff *t* verifies under a constant-time comparison and $N>N_{\mathrm{DEVEUI}}^{★}$. Following Reviewer 1’s request, we first give an informal analysis addressing each security property separately (Section 6.3.1), then a formal, game-based computational argument (Section 6.3.2), and finally a concise symbolic argument in the Dolev–Yao style introduced in Section 3 (Section 6.3.3). The three are complementary, not interchangeable: the informal analysis fixes vocabulary and scope, the computational argument justifies the numerical forgery bound, and the symbolic argument justifies the channel-level claims (interception, replay, reordering, injection) independently of any particular cryptographic assumption.

#### 6.3.1. Informal Security Analysis

1. **Origin authentication.** A message is accepted only if its tag verifies under the registered key for the claimed DEVEUI, so acceptance certifies possession of *K*, not physical device identity (point 5 below).
2. **Message integrity/tamper detection.** *P* binds DEVEUI, TIMESTAMP, *N*, and SENSOR together; changing any single bit of any field changes *P* and hence the required tag, so a modified-in-transit message fails verification except with the negligible probability quantified in Section 6.3.2.
3. **Freshness/replay protection.** Freshness is provided solely by the counter and the durable high-water mark ($N>N^{★}$), never by the timestamp (point 8); an exact retransmission of a previously accepted message therefore carries an already-seen *N* and is rejected.
4. **Counter reordering and skip behaviour.** The accept rule is $N>N^{★}$, not $N=N^{★}+1$, so out-of-order delivery of genuine messages is tolerated but not corrected: a captured higher-counter message delivered first is accepted and advances $N^{★}$, after which a genuine lower-counter message is rejected as stale. Section 4.2 and Table 1 work through this scenario in full; the property is a documented availability limitation (readings can be lost to reordering), not a forgery, because the attacker never causes acceptance of content it could not have captured from a genuine transmission.
5. ** Device-identity binding.** Binding DEVEUI into *P* prevents an attacker from relabelling a captured message as coming from a different device, but identity here is entirely a function of key possession: two devices provisioned with the same key would be cryptographically indistinguishable, which is why independent per-device keys (Section 4.5) are required for the compromise-containment property in point 7.
6. **Resistance to message forgery.** Without *K*, an adversary cannot compute a valid tag for a payload of its choosing; this is the subject of the formal argument in Section 6.3.2.
7. **Partial key compromise.** Because keys are independent per device, compromising *c* of *N* devices lets the adversary authenticate as exactly those *c* devices; under equal-rate devices and per-device rate limits the admitted forged traffic is bounded by the compromised devices’ traffic share c/N, with no such bound if rate limits are absent.
8. **Timestamp limitations.** TIMESTAMP is authenticated (tamper-evident, per point 2) and retained for logging and coarse sanity checks, but the server does not use it in the acceptance decision; consequently, the scheme needs no clock synchronisation, but also derives no freshness guarantee from the timestamp field at all—an attacker who replays or reorders a genuinely captured message changes nothing about its timestamp, since doing so would require recomputing the tag.
9. **Availability/denial-of-service limitations.** The scheme provides neither confidentiality nor availability: jamming, dropping, delaying, and reordering are all outside its scope (Section 3), and a large volume of forgery attempts, though cryptographically futile per point 6, still costs the server one verification per attempt, so gateway-level rate limiting is a necessary complementary control addressed separately from the cryptographic bound in Section 6.3.2.
10. **Physical compromise and key extraction.** The origin-authentication guarantee is conditional on key secrecy; extracting *K* from a device or the server’s key store fully compromises that device’s identity (the attacker becomes cryptographically indistinguishable from it), contained only to that device under independent per-device keys. The reference implementation does not evaluate secure boot, flash encryption, eFuse-protected storage, or a secure element, so physical-extraction resistance is an assumption, not a demonstrated property (Section 7).
11. **Canonical encoding.** The server rejects any representation of a message that is not byte-identical to the canonical form of Table 2 before computing a tag over it, so no two logically distinct field assignments can be made to collide into the same authenticated byte string under a looser parse; this closes a class of parser-level forgeries that is independent of the HMAC construction itself.
12. **Constant-time comparison.** The tag comparison is implemented using a constant-time routine (hash_equals), which is intended to prevent a straightforward byte-at-a-time timing side-channel on that specific comparison; this is narrower than a general side-channel-resistance claim, since power or electromagnetic leakage during the HMAC computation itself is not evaluated (Section 3).

#### 6.3.2. Formal Security Analysis: Existential Unforgeability Game

We phrase the forgery-resistance guarantee as a standard existential-unforgeability-under-chosen-message-attack (EUF-CMA) game, adapted to this scheme’s fixed message structure.

Adversary and oracle access. The adversary A does not know *K*. A may adaptively query a verification oracle with any bitstring and learn only whether it is accepted or rejected (and, if rejected, which check failed: format, tag, or counter—matching the reference implementation’s distinct rejection reasons); A may also passively observe polynomially many genuine, valid $\left(P_{i},t_{i}\right)$ pairs transmitted by the device, corresponding to real messages captured over the air. A never has access to a signing oracle beyond these captured pairs, since the device is not queryable (the scheme is unidirectional; Section 4.2).

Successful forgery. A wins if it submits some $M^{*}=t^{*}\left\|“|”\right\|P^{*}$ that the server accepts, where $P^{*}$ was never transmitted by the genuine device (a modified payload, a payload for a different DEVEUI under the same key, or any adversary-chosen content), or where $t^{*}$ verifies for a $P^{*}$ the device did transmit but $t^{*}$ was not the tag the device actually attached to it. Acceptance of an unmodified, previously-captured $\left(P_{i},t_{i}\right)$ pair with $N_{i}>N^{★}$ is explicitly excluded from this definition: it is a legitimate replay of authentic content under out-of-order delivery (point 4 above), not a forgery, and is treated separately by the freshness argument below.

Reduction. A successful forgery as defined above requires A to produce a valid $\left(P^{*},t^{*}\right)$ pair with $P^{*}$ not among the observed $\left(P_{i},t_{i}\right)$ pairs’ payloads. Under the assumption that HMAC-SHA-256 is a secure pseudorandom function and hence an unforgeable MAC [22,23], any adversary running in probabilistic polynomial time and succeeding at this with non-negligible probability could be used to construct a distinguisher or forger against the underlying PRF/MAC assumption, contradicting it; hence no such adversary exists except with negligible probability, conditioned on: the key *K* having sufficient entropy and being CSPRNG-generated and uncompromised; HMAC-SHA-256 being implemented correctly on both sides; the canonical encoding of point 11 above being unambiguous, so that no two distinct logical messages can be represented by the same authenticated byte string; and the tag comparison being constant-time (point 12), so that no partial-match information leaks through response timing.

Guessing bound and query/attempt budget. Independently of the reduction above, blind online guessing against an untruncated 256-bit tag succeeds with per-attempt probability at most $2^{-256}$, where one “attempt” is a single submitted message the adversary asks the server to verify. We define the adversary’s total query budget as *q*, the number of such attempts across the lifetime of the deployment; by a union bound the total forgery probability across the budget is at most $q\cdot 2^{-256}$, which remains negligible for any *q* a physically realisable adversary could mount (for example, $q=2^{64}$ leaves a bound of $2^{-192}$). This bound is purely cryptographic and is distinct from denial-of-service resource exhaustion: a large *q* costs the server one verification per attempt regardless of whether any attempt succeeds, so gateway-level rate limiting is a separate, complementary operational control against a brute-force stream saturating server capacity, and is not part of, nor a substitute for, the forgery-probability bound itself.

Freshness independent of the forgery argument. The counter/high-water-mark check operates independently of the argument above: even a $\left(P_{i},t_{i}\right)$ pair that is genuinely valid (because it was really transmitted by the device and hence trivially passes the EUF-CMA game, not being a forgery) is rejected if $N_{i}\leq N^{★}$, since $N^{★}$ is read, compared, and—on acceptance—advanced as a single durable, atomic operation (Section 4.5), so a server crash cannot leave an accepted message without its corresponding state update or vice versa. This is what makes exact replay of a captured message fail regardless of the cryptographic argument, and it is also why the timestamp, which is authenticated but never compared against a clock or a stored value, contributes nothing to freshness (point 8): only *N* and $N^{★}$ do.

Containment under key compromise. If the key-secrecy precondition of Section 3 is violated for one device, the argument above no longer applies to that device: an adversary holding its key can produce arbitrary valid $\left(P^{*},t^{*}\right)$ pairs for it by definition. Because keys are independent per device, this does not extend the adversary’s capability to any other device’s traffic; under equal-rate devices and per-device rate limits, the fraction of total traffic the adversary can forge is bounded by the compromised devices’ share c/N (point 7).

#### 6.3.3. Symbolic (Dolev–Yao) Argument

To complement the computational argument above with the channel-level reasoning standard for authentication protocols, we give a concise symbolic argument in the Dolev–Yao style [32] introduced in Section 3. The attacker is the communication channel: it can intercept every message; replay any previously observed message verbatim; reorder or delay delivery arbitrarily; inject arbitrary messages of its own construction; and modify any message it intercepts before re-injecting it. The attacker does not learn *K* (the key-secrecy precondition). Within this symbolic abstraction, HMAC-SHA-256(K,·) is treated as an ideal function the attacker cannot invert or evaluate without *K*; consequently, the attacker cannot construct a valid tag for any payload $P^{*}$ it did not observe already correctly tagged by the genuine device—symbolically justifying the same non-forgeability conclusion the computational argument reaches by a cryptographic reduction. A message the attacker replays verbatim is rejected if its counter is not strictly greater than the durably stored high-water mark, matching the freshness argument above; a message the attacker has genuinely captured but not yet delivered, if it carries a counter higher than anything yet accepted, may be accepted when the attacker chooses to deliver it, which is an availability/ordering limitation the symbolic model makes explicit rather than a forgery, since the delivered content is exactly what the device produced. The symbolic model does not, by itself, quantify the probability of guessing a valid tag (that is the role of the computational argument in Section 6.3.2) and does not model resource-bounded denial-of-service behaviour; it is used here only to state precisely which channel-level manipulations the scheme tolerates and which it does not, matching Section 3’s CAN/CANNOT lists.

## 7. Limitations and Threats to Validity

The scheme provides neither confidentiality nor availability by design; an adversary who can jam, drop, delay, or reorder frames can cause data loss (never forgery), and defences against that—redundant paths, server-side gap and rate monitoring—are complementary and out of scope. The origin-authentication guarantee is conditional on key secrecy: extracting a device’s key fully compromises that device’s identity, contained only to the affected device under independent keys and per-device rate limits.

On evaluation, the static code footprint is measured on the ESP32-S3 Xtensa instruction-set architecture (compiled, not board-timed) and device–server tag agreement is verified, but per-message runtime cycles and energy are not reported as numeric estimates in this revision, since we could not identify an architecture-matched, citable source for a software SHA-256 cycles-per-byte rate on this core (Section 5.1); board-level timing and energy remain future work. The poisoning experiments use ten independent seeds and a single learner (tree-based gradient-boosted regression) on two datasets, so they support cross-dataset but not cross-model external validity, and the online-learning regime, where retraining and poisoning interact over time, is not covered. Poison is inserted into the chronological training stream as in-distribution or filter-aware readings with realistic (timestamp-derived) seasonal features, and the attacker experiments cover forgery, tampering, replay, stealthy and filter-aware poisoning, and partial key compromise, but not an adaptive side-channel adversary. The reference implementation uses placeholder keys and none of the platform hardening (secure boot, flash encryption, encrypted or eFuse-protected storage, secure element) a deployment would need, so physical-extraction resistance and secure provisioning are assumed, not demonstrated, and cryptographic key rotation itself is not implemented (though the counter-state side of re-provisioning is, and is tested; Section 4.5). Full reproduction requires the Supplementary Materials, which provides the firmware and server code, parser rules, test-vector and attacker scripts, poisoning-study scripts and raw result tables, static-footprint commands and outputs, and the airtime/energy and NVS-endurance calculations.

## 8. Conclusions

We have presented a lightweight, unidirectional, encryption-free authentication scheme for raw or custom LoRa telemetry—links that, unlike a correctly configured LoRaWAN network, carry no built-in message integrity or replay protection. Keying an HMAC-SHA-256 tag with a per-device secret over the device identifier, a timestamp, a monotonic counter, and the sensor value delivers origin authentication, tamper evidence, and duplicate/stale rejection under a server high-water mark, with no encryption, return channel, or clock synchronisation. Under key secrecy, unauthenticated forged rows are rejected before training whatever values they carry; where LoRaWAN is in use, the scheme is a complementary application-layer check independent of the network-layer MIC, not a replacement.

The main contribution is engineering rather than new cryptography: a standard MAC construction is made precise, implemented consistently, audited, and costed on the target instruction-set architecture. The audit showed how easily a plausible design fails in practice—a modulus computed with the C bitwise-XOR operator, mismatched truncation between device and server, an instruction count taken on the wrong architecture, and (found in this revision) a counter convention that let the firmware transmit a first message the server’s own stated rule would reject—each silently weakened the original scheme, so correcting these and re-measuring on the actual ESP32-S3 ISA matters as much as the design. The dominant quantified added cost is transmitting the tag, which raw-binary encoding would roughly halve; we make no claim about HMAC computation’s relative cost, since board-level HMAC runtime and energy were not measured in this work. The poisoning study, now run over ten seeds with training-only statistics and forged readings inserted into the chronological stream, is a motivating illustration of why provenance checks should occur before value-based cleaning: for the single model tested here, a robust value filter discarded the genuine extremes that matter and could be steered by a distribution-aware attacker, whereas authentication rejected forged rows before training regardless of how the poisoning was constructed. The central lesson is practical: standard HMAC authentication is only useful if the byte encoding, key handling, replay state, server parser, and implementation measurements are correct.

Deployment still requires protected key storage, secure provisioning, rotation, availability engineering, and optionally reduced tag overhead. A stronger evaluation would pair this engineering baseline with optimization-based regression attacks and a wider range of poison-construction strategies; that evaluation is left for future work, alongside on-board energy measurement, more model families and the online-learning setting, an adaptive side-channel adversary, and fleet-scale key management. We regard the scheme as a small, verifiable building block on which such extensions can rest.

## Figures and Tables

**Table 1:** Worked example of the reordering scenario. The server’s high-water mark $N^{★}$ starts at 9 (some earlier reading was the last one accepted). The device then produces three genuine consecutive readings, N=10,11,12, which an in-range attacker captures before any of them reaches the server and delivers out of order. Every payload and tag shown for N=10,11,12 is byte-identical to what the device actually transmitted; only the delivered order is under attacker control.

Step	Delivered Message	Tag Valid?	$N^{★}$ Before	Decision	Why
1	attacker forwards captured N=12 first	yes (genuine, untouched)	9	ACCEPT (12>9); $N^{★}\;\leftarrow \;12$	Genuinely produced by the device; not previously accepted.
2	attacker later forwards captured N=10	yes (genuine, untouched)	12	REJECT (stale; 10≤12)	Authentic but no longer fresh: a lower counter than what is already accepted.
3	attacker later forwards captured N=11	yes (genuine, untouched)	12	REJECT (stale; 11≤12)	Same reason; the reading is genuinely lost to the pipeline, not forged.
4	attacker edits the N=12 payload’s SENSOR field before forwarding	no (HMAC mismatch)	12	REJECT (bad-tag)	Any change to *P* changes the required tag; without *K* the attacker cannot recompute a matching one.

**Table 2:** Canonical field encoding. All fields are joined by the ASCII delimiter “|”; the HMAC is taken over the raw UTF-8 bytes of the joined payload. *SENSOR* range is a deployment-policy choice enforced by the reference server parser, not a constraint of the wire format itself.

Field	Encoding
DEVEUI	8-byte identifier as exactly 16 lowercase hex characters, MSB first
TIMESTAMP	unsigned 64-bit Unix time (UTC seconds), base-10, no leading zeros; the canonical format permits any value in $\left[0,2^{64}-1\right]$, including 0 (Unix epoch), since the timestamp is not used in the acceptance decision (Section 4.5)
*N*	32-bit monotonic counter, base-10 unsigned, no leading zeros
SENSOR	signed base-10 integer in deci-degrees Celsius (e.g., 23.5 °C → 235); the reference server parser enforces a deployment-policy range of −100.0 °C to +200.0 °C (−1000 to 2000), which is not part of the canonical wire format and may be set differently per deployment
TAG	HMAC-SHA-256 output, 64 lowercase hex characters (64 ASCII bytes)

**Table 3:** Per-message cost summary. Static code size is measured; airtime is calculated from the LoRa parameters; transmit energy uses $E_{\mathrm{TX}}=V\cdot I_{\mathrm{TX}}\cdot t_{\mathrm{air}}$ with example values at $I_{\mathrm{TX}}=32$ mA (SX1262 low-output-power end under DC-DC regulation), V=3.3 V. Sizes are given in KiB (1024 bytes); the 16 MB flash capacity is the manufacturer’s decimal-MB rating. Board-level timing and energy were not measured, and no numeric CPU-cycle/energy-per-HMAC estimate is reported in this revision (see Section 5.1).

Metric	Value	Basis
.text (SHA-256/HMAC)	1572 bytes (≈1.54 KiB)	measured
Static instruction count	540 Xtensa instructions (hashBlock: 145)	measured
.rodata (SHA-256/HMAC)	288 bytes (≈0.28 KiB)	measured
Stack use, no heap	≈400 bytes (≈0.39 KiB)	estimated
Whole-firmware flash increase	≈1.9 KiB (of 16 MB)	measured (linked)
SHA-256 compression blocks/HMAC	4 (256 bytes through compression)	derived
Unauthenticated/authenticated message size	34/99 bytes (2.91×, +191%)	measured (format)
Unauthenticated/authenticated airtime	77.1/169.2 ms (2.20×, +120%)	calculated
Incremental airtime (tag)	92.2 ms	calculated
Example added TX energy (tag)	≈9.7 mJ	example (E=VIt)

**Table 4:** Cost comparison with existing authentication approaches. Superscript letters key to the notes below the table, which carry the caveats needed to interpret each row correctly.

Scheme	Mechanism	Added Bytes	Added Airtime	Storage/Device	Evidence
This work	HMAC-SHA-256	+65 B (2.91×)	+92.2 ms (2.20×)	HWM (4 B) + key (32 B)	Measured/calculated
LoRaWAN MIC ^a^	AES-CMAC	+4 B ^b^	n/e	counter state	Literature (spec)
ECDH ^c^	Key exchange	n/e	n/e	key-dependent	Qualitative only
Generic AEAD ^d^	Enc. + integrity	n/e	n/e	1 key/device	Qualitative only

n/e = not established (no measurement or comparable literature figure reported here). HWM = high-water mark. ^a^ [18]. LoRaWAN’s MIC operates at a different protocol layer (the network-layer MAC frame) than the application-layer tag in this work; its overhead is reported for reference, not as a directly subtractable like-for-like number (Section 2). ^b^ 32-bit (4-byte) AES-CMAC MIC, spec-mandated, computed over the whole MAC frame; the 32-bit frame counter’s 16 least-significant bits are transmitted on the air interface. ^c^ [20]. A previously cited peak-memory figure was measured on a different toolchain/platform; no like-for-like number is reported here (Section 5.1). ^d^ e.g., AES-GCM at the application layer; not implemented or measured in this work.

**Table 5:** Observed server behaviour in the implementation test, for genuine and adversarial message classes. Counts are outcomes on this finite test set, not an estimate of the cryptographic forgery probability.

Message Class	Count	Accepted by Server
Genuine	5000	5000 (100%)
Random tag	5000	0 (0%)
Wrong key	5000	0 (0%)
Tampered payload	5000	0 (0%)
Replay	5000	0 (0%)

## Data Availability

The poisoning experiment uses two publicly available real environmental time series: daily minimum temperatures for Melbourne [38] and hourly Beijing PM2.5 air-quality readings [41]. The adversarial poisoned rows are synthetically generated by the attack profiles described in Section 6.2. The generating scripts, firmware and server code, and raw results supporting the reported findings are included in the reproducibility archive submitted as Supplementary Materials with this manuscript. The archive will be deposited in a permanent repository before publication.

## Supplementary Materials

The following supporting information can be downloaded at https://www.mdpi.com/article/10.3390/s26196276/s1, A reproducibility archive is submitted with this manuscript as Supplementary Materials. It contains the firmware and server verifier, parser rules, test-vector scripts and outputs, poisoning-study scripts and raw results, plotting scripts, static-footprint outputs, airtime/example-energy calculations, NVS-endurance estimate, dataset information, and a README with reproduction commands. The archive will be deposited in a permanent repository before publication. Board-level HMAC timing and energy are not measured, and no numeric CPU-cycle/energy-per-HMAC estimate is reported in this revision; see Section 5.1 for what is measured, calculated, and explicitly not quantified.

## Abbreviations

The following abbreviations are used in this manuscript:

IoT	Internet of Things
HMAC	Hash-based Message Authentication Code
MAC	Message Authentication Code
SHA	Secure Hash Algorithm
TOTP	Time-based One-Time Password
HKDF	HMAC-based Key Derivation Function
RTC	Real-Time Clock
RMSE	Root-Mean-Square Error
LoRa	Long Range (radio modulation)
DEVEUI	Device Extended Unique Identifier
NVS	Non-Volatile Storage
MIC	Message Integrity Code
CSPRNG	Cryptographically Secure Pseudo-Random Number Generator
HSM	Hardware Security Module
KMS	Key Management System
PRF	Pseudorandom Function
AEAD	Authenticated Encryption with Associated Data
ISA	Instruction-Set Architecture
DY	Dolev–Yao (symbolic adversary model)
eCK	extended Canetti–Krawczyk (authenticated-key-exchange security model)
EUF-CMA	Existential Unforgeability under Chosen-Message Attack
